# WNT16 elevation induced cell senescence of osteoblasts in ankylosing spondylitis

**DOI:** 10.1186/s13075-021-02670-0

**Published:** 2021-12-08

**Authors:** Sungsin Jo, Subin Weon, Bora Nam, Mi-Ae Jang, Hyundeok Kang, Tae-Jong Kim, Ye-Soo Park, Tae-Hwan Kim

**Affiliations:** 1 Hanyang University Institute for Rheumatology Research, Seoul, 04763 Republic of Korea; 2 Department of Translational Medicine, Graduate School of Biomedical Science and Engineering, Hanyang University, Seoul, 04763 Republic of Korea; 3 Department of Rheumatology, Hanyang University Hospital for Rheumatic Disease, 222-1, Wangsimni-ro, Seongdong-gu, Seoul, 04763 Republic of Korea; 4 Department of Laboratory Medicine and Genetics, Soonchunhyang University Bucheon Hospital, Soonhchunhyang University College of Medicine, Bucheon, 14584 Korea; 5 Department of Biomedical Systems Informatics, Brain Korea 21 PLUS project for Medical Science, Yonsei University College of Medicine, Seoul, 03722 Republic of Korea; 6 Deparment of Rheumatology, Chonnam National University Medical School and Hospital, Gwangju, 61469 Republic of Korea; 7 Department of Orthopedic Surgery, Guri Hospital, Hanyang University College of Medicine, Guri, 11923 Republic of Korea

## Abstract

**Background:**

WNT16 is critical for bone homeostasis, but the effect of WNT16 in ankylosing spondylitis (AS) is still unknown. Here, we investigated whether WNT16 influences bone formation and pathophysiological changes of AS in an in vitro model.

**Methods:**

The bone tissue from the facet joints was obtained from seven disease control and seven AS patients. Primary osteoprogenitor cells of the facet joints were isolated using an outgrowth method. Isolated osteoprogenitor cells from both control and AS tissues were analyzed by microarray, RT-qPCR, immunoblotting, and immunohistochemistry. The bone-forming activity of osteoprogenitor cells was assessed by various in vitro assays. β-galactosidase staining and senescence-associated secretory phenotype (SASP) using RT-qPCR were used to assess cell senescence.

**Results:**

In microarray analysis, WNT16 expression was significantly elevated in AS osteoprogenitor cells compared to the control. We also validated that WNT16 expression was elevated in AS-osteoprogenitor cells and human AS-bone tissues. WNT16 treatment inhibited bone formation in AS-osteoprogenitor cells but not in the control. Intriguingly, AS-osteoprogenitor cells were stained markedly with β-galactosidase for cell senescence in WNT16 treatment. Furthermore, in an H_2_O_2_ stress-induced premature senescence condition, WNT16 treatment increased cell senescence in AS-osteoprogenitor cells and WNT16 treatment under the H_2_O_2_ stress condition showed an increase in p21 protein and SASP mRNA expression. The WNT16-induced SASP expression in AS-osteoprogenitor cells was reduced in WNT16 knockdown cultures.

**Conclusion:**

WNT16 is highly expressed in AS and WNT16 treatment facilitated cell senescence in AS-osteoprogenitor cells during osteoblast differentiation accompanied by suppression of bone formation. The identified role of WNT16 in AS could influence bone loss in AS patients.

**Supplementary Information:**

The online version contains supplementary material available at 10.1186/s13075-021-02670-0.

## Background

Ankylosing spondylitis (AS) is a chronic inflammatory arthritis characterized by spinal ankylosis due to excessive bone formation [1–3]. Clinically, AS has shown a paradoxical feature including bone loss as well as bone formation simultaneously [4–10]. Pathological bone changes in AS have been associated with the Wnt signaling pathway [11–13] raising intriguing questions of the molecular partners of Wnt signaling and bone changes in AS. Among the Wnt molecules, many studies have clearly shown that WNT16 signaling increases bone formation while WNT16 inhibition leads to decreased bone formation [14–16]. Although the role of WNT16 signaling and bone formation has been studied using various genetic murine models, the conclusions in human AS are still elusive.

The main cause of bone loss in patients with AS is unclear. It has been reported that the prevalence of AS patients with bone loss is about 25% and low vertebral bone mineral density in AS is closely associated with high modified Stoke Ankylosing Spondylitis Spinal Score (mSASSS) and C-reactive protein (CRP) [17, 18]. Chronic inflammation and immobilization have been speculated as risk factors in bone loss in AS patients. A recent study reported increased cellular senescence in mesenchymal stem cells (MSCs) derived from the bone marrow of patients with AS [19]. In this paper, oxidative stress (OS) facilitated senescence by an increase in the release of reactive oxygen species (ROS) and mitochondrial deregulation. Clinically, oxidative stress status and associated serum proteins were higher in AS and associated with low bone mineral density in patients with AS [20–22]. However, whether the senescence influences bone-forming activity in AS is still unknown.

Elevated expression of Wnt/β-catenin signaling has been reported to be associated with AS pathophysiology. Wnt molecules have been studied a lot with bone formation, but little is known about the association of Wnt with osteoporotic bone loss in AS. In the present study, we evaluated whether WNT16 could regulate bone formation and bone loss in AS. Moreover, the physiological changes caused by WNT16 were examined in AS-osteoprogenitor cells.

## Materials and methods

### Human facet joint bone tissue

The present study was conducted in accordance with the Helsinki Declaration and approved by the Institutional Review Board of Hanyang University, Seoul (IRB 2014-05-002), and Guri Hospital (IRB 2014-05-001). We obtained facet joints from seven AS patients who met modified New York criteria [23, 24] and underwent spinal surgery and from seven patients with non-inflammatory disease as the control. Clinical demographics are shown in Table 1.

**Table 1:** Clinical demographics in this study

	Bone tissue donor		Serum donor	
	disease controls (*n*=7)	AS patients (*n*=7)	healthy controls (*n*=30)	AS patients (*n*=36)
Age, year	54.1 (59–46)	43.4 (37–47)	31.0 (28.0–36.3)	54.4 (44.3–60.9)
Male sex	7, 100%	7, 100%	30, 100%	36, 100%
HLA-B27 positivity	0, 0%	7, 100%	30, 0%	28, 100% (*n*=28)
Symptom duration, year	N/A	14.5 (7.0–22.0)	N/A	19.6 (11.4–25.2)
mSASSS	N/A	N/A	N/A	33.4 (9.0–55.8)
CRP	N/A	0.4 (0.4–2.4)	N/A	0.4 (0.4–1.0) (*n*=35)
Spinal BMD	N/A	N/A	N/A	1.07 (0.9–1.3)
Spinal T-score	N/A	N/A	N/A	0.35 (−0.7–2.4)
Use of TNF inhibitors	0, 0%	4, 57.1%	0, 0%	24, 66.7%

### Isolation of osteoprogenitor cells

The protocol for the isolation of osteoprogenitor cells was previously reported [25–28]. Briefly, osteoprogenitor cells were isolated until the fourth passage using the outgrowth method for one month in Dulbecco’s modified Eagle medium-high glucose (DMEM-HG; Hyclone, SH30243.01) containing 10% fetal bovine serum (FBS; Gibco, 16000-044) and 1% antibiotics (penicillin/streptomycin; Gibco, 1540-122). All isolated osteoprogenitor cells were checked for mycoplasma using a PCR-based method (Takara, 6601) before long-term storage in liquid nitrogen. Passages 3~7 were selected and used for further experiments.

### Microarray analysis

Passage 3 of control- and AS-osteoprogenitors was used and performed using the Affymetrix GeneChip Human Gene 2.0 ST array. For microarray analysis, background adjustment, normalization, and median polish summarization of CEL files were performed with the robust multi-average method using Partek Genomics Suite software version 7.0. Differential expression was calculated from the normalized data using ANOVA and visualized as a heatmap for Wnt genes.

### RT-qPCR

Primary osteoprogenitor cells were lysed using TRIzol, and 1.0 μg of the total RNA was subjected to reverse transcription using a cDNA kit (Thermo Fisher Scientific, Waltham, MA, USA). Relative gene transcript levels were determined by RT-qPCR using SYBR Green, and qPCR was performed in triplicate for each sample. The mean expression level of each gene was normalized to GAPDH. The primers used in the study are listed in Table 2.

**Table 2:** RT-qPCR primers

Gene	5′-Forward-3′	3′-Reverse-5′
GAPDH	CAAGATCATCAGCAATGCC	CTGTGGTCATGAGTCCTTCC
WNT16	CTACGGAGCCCAAGGAAACT	CTCTCGTGTCTGAACTGGCT
TP53	ACCTATGGAAACTACTTCCTGAAA	CTGGCATTCTGGGAGCTTCA
CDKN1A (p21)	GCAGAGGAAGACCATGTGGA	CGGCGTTTGGAGTGGTAGAA
CASP3	TCATTATTCAGGCCTGCCGT	GTCGGCCTCCACTGGTATTT
IL1A	TGGTAGTAGCAACCAACGGG	GGTGCTGACCTAGGCTTGAT
IL1B	CAGAAGTACCTGAGCTCGCC	AGATTCGTAGCTGGATGCCG
IL6	ATGAACTCCTTCTCCACAAGCG	CTCCTTTCTCAGGGCTGAG
IL8	TTCTGCAGCTCTGTGTGAAGG	TAATTTCTGTGTTGGCGCAGTG

### Immunoblot assays

Stimulated osteoprogenitor cells were lysed with 1X RIPA buffer containing phosphatase (5870S, Cell Signaling Technology, USA) and protease (535140, Calbiochem, USA) inhibitors. Proteins were quantified using the Bradford assay. Total cell protein (10–30 μg) was subjected to immunoblotting using the following antibodies: β-catenin (9562S), active β-catenin (19807S), phos-AKT (Ser473) (4060), total-AKT (4691), phos-ERK (9101), total-ERK (9102), and GAPDH (2118) from Cell Signaling Technology (Danvers, MA, USA). CDKN1A (p21; A19094) and OCN (ab13420) antibodies were obtained from Abaclonal and abcam, respectively. WNT16 (sc-271897), CASP3 (sc-7272), and OPN (sc-21742) antibodies were obtained from Santa Cruz Biotechnology. Goat anti-rabbit IgG (111-035-003) and goat anti-mouse IgG (115-035-003) secondary antibodies were obtained from Jackson ImmunoResearch (West Grove, PA, USA).

### Cell viability

The water-soluble tetrazolium salt (WST) assay was performed using the EZ-CYTOX kit (Dogen Bio, EZ-1000) according to the manufacturer’s instructions. Primary osteoprogenitor cells were seeded into 96-well plates at 1×10^3^ cells/well and treated with WNT16 at the indicated dose and time point (days). At the indicated day, 10 μl WST solution was added to the cells and incubated for 1 h. Absorbance at 450 nm was measured using a microplate reader (Thermo Fisher Scientific, Inc.).

### Immunohistochemistry

The detailed materials and methods for immunohistochemistry (IHC) analysis were previously described [25]. Briefly, facet joint tissues were fixed in 10% formalin for a week, decalcified with 10% formic acid for a week, and embedded in paraffin. Tissue slides (5 μm) were treated with Neo-Clear for deparaffination, followed by dehydration with ethanol solutions (100 to 50% of ethanol), permeabilization with 0.25% Triton X-100, and blocking with BLOXALL (Vector Lab, SP-6000) for 1 h. The slides were then incubated with primary WNT16 antibodies (sc271897, Santa Cruz Biotechnology; 1:100 ratio in antibody diluent) overnight, followed by washing with PBS-T, incubation with biotinylated secondary antibodies for 1 h, washing with PBS-T, incubation with ABC kit components as specified by the manufacturer (Vector Lab, PK-6102), and incubation using a DAB substrate kit (Vector Lab, sk4100) for 1 to 5 min. The slides were counterstained with haematoxylin (Merck, 1.05174.0500) for 10 s, dehydrated though ethanol and Neo-Clear, and coverslipped using a permanent mounting medium (Vector Lab, H-5000). Microscopy was used to visualize and image stain cells (Leica).

### Human WNT16 serum level measurements

Sera were collected from 30 healthy male donors and 36 patients with AS who met the modified New York criteria [23]. Serum collection methods and storage conditions were previously described [25]. We measured WNT16 serum level using a human WNT16 ELISA kit (CUSABIO, CSB-EL026132HU) according to the manufacturer’s instructions.

### In vitro bone formation

The protocol for the use of osteoprogenitor cells for assessing bone-forming activity was previously reported [25–28]. Briefly, osteoprogenitor cells were seeded in 96-well plates at 1×10^4^ cells/well and incubated for 24 h. These cells were changed to osteogenic medium containing DMEM-HG (Hyclone, SH30243.01) containing 10% fetal bovine serum (FBS; Gibco, 16000-044), 1% antibiotics (penicillin/streptomycin; Gibco, 1540-122), 50 μM ascorbic acid (Sigma, A4544), 10 mM β-glycerol phosphate (Santa Cruz Biotechnology, sc-220452A;), and 100 nM dexamethasone (Sigma, D2915). The medium was changed every 3 or 4 days and bone-forming activity was assessed at the indicated days. The matrix maturation of bone formation was assessed by alkaline phosphatase (ALP) activity (BioVision, K412-500), ALP (Sigma, 85L2), and collagen using Picro-Sirius Red (Abcam, ab150681) staining. The matrix mineralization of bone formation was assessed using Alizarin Red S (ARS; ScienCell, 8678), hydroxyapatite (HA; Lonza, PA-1503), and Von Kossa (5% silver nitrate solution; SAMCHUN Chemical, S0228) staining. Stained wells were imaged via microscopy (Nikon eclipse Ti-U, Nikon). The overall image of a well for 96 well plates and its enlarged changes were seen in Suppl. Figure 1.

In the quantification of bone mineralization, we followed the manufacturer’s instructions for ARS and HA staining. To analyze Von Kossa staining, each image was manually imported to the ImageJ, defined the area by CIRCLE or SQUARE; the image was segmented and then put on the “Analyze” and “Measure” to obtain the area percentage of each pre-defined class. Before calculation, the VON image value at day 0 was also prepared in the same way as above. Next, we collected the “Area” and “Percent” values, put them in Excel, and calculated the Relative Density values for the VON image at day 0 as a control.

### Promoter assays

SaOS2 cells (Korean Cell Line Bank, 30085) were seeded in 60 mm plates at 2×10^5^ cells/well and co-transfected with each alkaline phosphatase (ALP), osteoblast-specific element (OSE), bone sialoprotein (BSP), osteocalcin (OCN), TOP, or FOP promoter DNA (3 μg/well) and *Renilla* luciferase (0.3 μg/well) using Lipofectamine 3000 (Thermo Fisher, L3000015). A day after transfection, the cells were reseeded in 12-well plates at 5×10^4^ cells/well and incubated for 24 h. The next day, cells were treated with WNT16 (50 ng/mL) for 24 h and lysed to measure luciferase activity (Promega, E1500), according to the manufacturer’s instructions. The luciferase activity data were normalized to *Renilla* luciferase activity.

### Small interference RNA (siRNA) and knockdown efficiency testing

Five siRNA oligos against human WNT16 were designed and generated by GENOLUTION Inc. (Seoul, Korea). Oligo sequences for siRNA are listed in Table 3. To test WNT16 knockdown efficiency, FOB cells (ATCC, CRL-11372) were seeded in 6-well plates and transfected with one of the five siRNA oligos against WNT16 using Lipofectamine 3000 (Thermo Fisher, L3000015). After 48 h, the transfected FOB cells were lysed and analyzed by RT-PCR (Suppl. Figure 2). Selected siRNA oligos were used for further investigation.

**Table 3:** siRNA oligos against WNT16

siRNA	Sequence (5′-3′)	
Negative control	Sense	CCUCGUGCCGUUCCAUCAGGUAGUU
	Antisense	CUACCUGAUGGAACGGCACGAGGUU
WNT16 #1	Sense	CCAAGUUGAUGUCAGUAGAUU
	Antisense	UCUACUGACAUCAACUUGGUU
WNT16 #2	Sense	GGAUGAUCUGCUCUAUGUUUU
	Antisense	AACAUAGAGCAGAUCAUCCUU
WNT16 #3	Sense	CCAACUACUGUGUAGAAGAUU
	Antisense	UCUUCUACACAGUAGUUGGUU
WNT16 #4	Sense	CUGAUCAACCCAUCAAUCAUU
	Antisense	UGAUUGAUGGGUUGAUCAGUU
WNT16 #5	Sense	CUGACUUACCCUUUCAUGUUU
	Antisense	ACAUGAAAGGGUAAGUCAGUU

### Senescence staining with β-galactosidase

To test the effect of WNT16 on cell senescence, AS-osteoprogenitor cells were seeded in 96-well plates at 4×10^3^ cells/well and incubated for 24 h. The next day, the cells were stimulated H_2_O_2_ (200 μM) for 2 h, washed with 1X PBS, and suspended in new growth medium with WNT16 (50 ng/mL) or vehicle. After 3 days, the senescence effect of WNT16 was assessed using a senescence detection kit (BioVision, K320), according to the manufacturer’s instructions. After staining, stained wells were imaged by microscopy (Nikon eclipse Ti-U). For senescence quantification, stained images were analyzed by counting senescence positive cells.

### Statistical analysis

Data images were generated using GraphPad Prism 6 software (GraphPad Software, Inc.). Two-tailed Student’s *t* tests were used to compare data between two unpaired groups. All data are expressed as the median and interquartile range (*n*≥3). A *p*<0.05 was considered statistically significant.

## Results

### WNT16 is highly expressed in the facet joint of AS

**Fig. 1:**
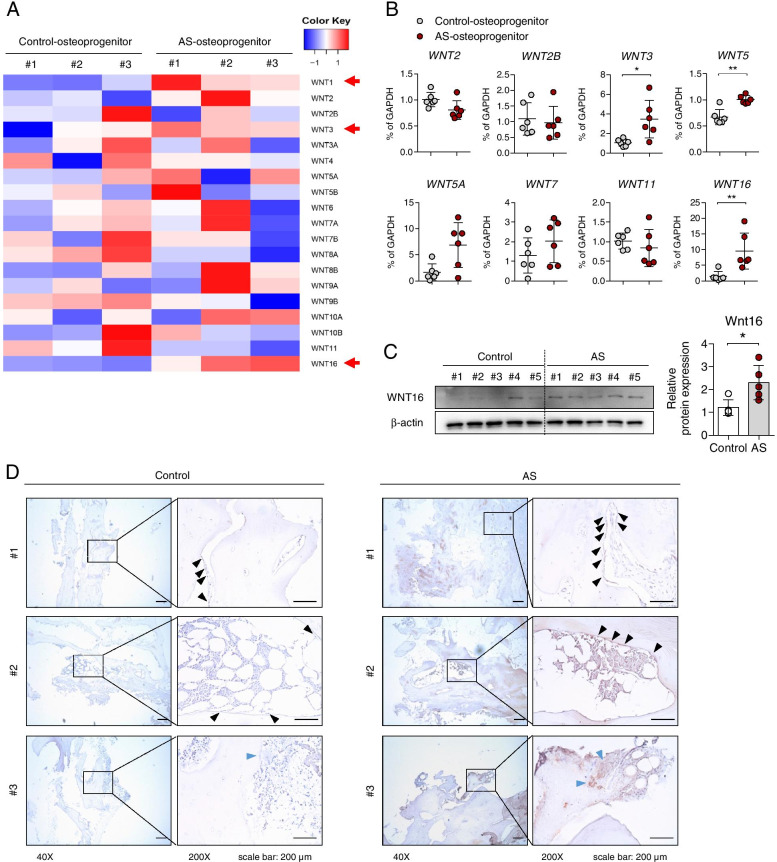
WNT16 is highly expressed in facet joint of AS. **A** Heatmap diagram of differentially expressed probes mapped to Wnt genes in AS and control osteoprogenitor cells. In AS osteoprogenitor cells, WNT16 showed significant upregulated expression with a *p* value < 0.05 and fold change > 2 compared to the control (*n*=3 per each group). **B** Verification of mRNA level using qPCR of **A** for the Wnt family (*n*=6 per each group). **C** Immunoblotting for WNT16 protein level in control and AS-osteoprogenitor cells (*n*=5 per each group). **D** Immunohistochemistry (IHC) staining of WNT16 in spinal facet joint tissue from control or AS patients. Black arrows indicate bone-lining cells, and blue arrows indicate periosteum in facet joints tissues. Three representative images are shown (*n*=7 per each group). Data are presented as the median and interquartile range. *p<0.05, **p<0.01

We performed microarray experiments with control and AS-osteoprogenitor cells and analyzed the data to attain molecular insights among the Wnt genes in those samples. Differential expression gene (DEG) analysis revealed that WNT1, WNT3, and WNT16 were highly expressed in AS-osteoprogenitor cells (Fig. 1A, red arrows). We validated these data using RT-qPCR and showed that WNT1 was not detected, but WNT3, WNT5, and WNT16 were significantly increased in AS (Fig. 1B). Since the functional role of WNT3 and WNT5 in AS had been reported elsewhere [3], we focused the role of WNT16 in AS-osteoprogenitor cells. WNT16 protein was found to be higher in AS-osteoprogenitor cells relative to the control, which was revealed by immunoblotting and its quantification (Fig. 1C). We confirmed that there was no significant difference in WNT16 serum levels between healthy and AS groups (Suppl. Figure 3), but we consistently found increased WNT16 expression in bone-lining cells (black arrows) and in the facet joint periosteum tissue (blue arrows) in AS-osteoprogenitor cells compared with the control (Fig. 1D). Collectively, WNT16 was significantly elevated in AS-osteoprogenitor cells and facet joint of AS.

### WNT16 treatment decreased matrix mineralization during osteoblast differentiation and increased senescence in AS

**Fig. 2:**
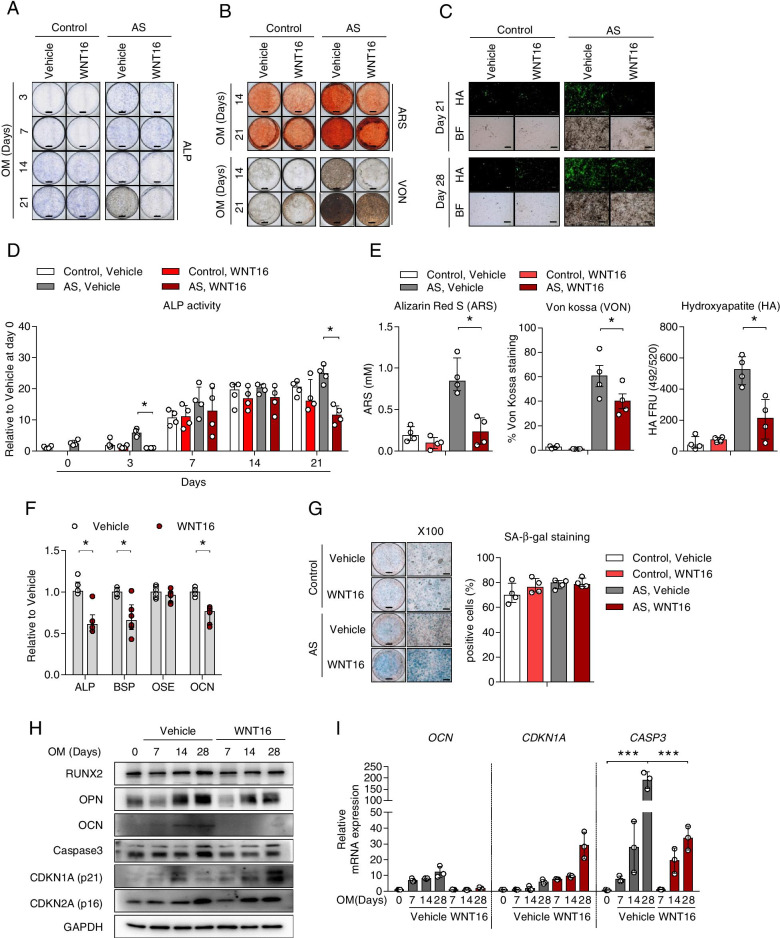
WNT16 treatment inhibited mineralization and promoted cell senescence of AS-osteoprogenitor cells during osteoblast differentiation. Both control and AS-osteoprogenitor cells were differentiated with osteogenic medium in the presence of vehicle or WNT16. Analysis of **A** ALP staining, **B** ARS staining, and VON staining, **C** HA staining, **D** intercellular ALP activity at indicated day, **E** quantitation of ARS and VON at day 21, and HA staining at day 28 (*n*=4 per each group). **F** SaOS2 cells were co-transfected with 3 μg ALP, BSP, OSE, or OCN promoter plasmid along with β-gal plasmid for 48 h followed by and WNT16 treatment for 24 h and then analyzed by luciferase assay (*n*=6 per each group). **G** SA-β-gal staining was performed at 28 days of osteoblast differentiation of control and AS-osteoprogenitor cells. Counting results of Fig. 3G (right panel) (*n*=4 per each group). AS-osteoprogenitor cells were differentiated into mature osteoblasts in the presence of vehicle and WNT16 (50ng/ml). Analysis of **H** immunoblotting for protein level and **I** RT-qPCR for mRNA level (*n*=3 per each group). Scale bar = 200 μm. Data are presented as the median and interquartile range. *p<0.05

We next investigated whether WNT16 treatment influences matrix maturation and mineralization of osteoprogenitor cells during osteogenic differentiation. Two doses (25 and 50 ng/ml) of WNT16 did not affect the proliferation rate of AS-osteoprogenitor cells (Suppl. Figure 4), but treatment with 50 ng/mL WNT16 inhibited ALP activity and staining status in AS-osteoprogenitor cells. To investigate the effect of WNT16, both control and AS-osteoprogenitor cells were treated with 50 ng/mL WNT16 during osteoblast differentiation. As shown in Fig. 2A–E, treatment with WNT16 significantly inhibited bone matrix maturation (ALP staining and activity) and matrix mineralization (ARS, VON, and HA staining) in AS-osteoprogenitor during osteogenic differentiation. There is no substantial observation in control-osteoprogenitor, but these inhibitory effects by WNT16 were pronounced in AS-osteoprogenitor cells. WNT16 treatment also reduced bone formation-related gene promoter activity in alkaline phosphatase (ALP), bone sialoprotein (BSP), and osteocalcin (OCN), as revealed by luciferase activity (Fig. 2F). Promoter activity of osteoblast-specific element (OSE) and mRNA expression of Osterix were no substantial changes in WNT16 treatment (data not shown). Intriguingly, AS-osteoprogenitor cells were markedly stained with SA-β-gal at 28 days of differentiation when compared with the control although the cell staining rates were not significantly different (Fig. 2G). As shown in the 2H, caspase 3 protein was upregulated in the vehicle group of AS-osteoprogenitor cells accompanied by gain of bone formation-related protein such as OCN and OPN during osteogenic differentiation, whereas the WNT16 treatment group inhibited the upregulation of those expressions and promoted CDKN1A (called as p21, cell cycle regulator as wells senescence indicator) expression in AS-osteoprogenitor. The RT-qPCR results of OCN, CDKN1A, and CASP3 were consistent with Fig. 2H (Fig. 2I). These findings show that treatment with WNT16 inhibits matrix maturation and mineralization during osteogenic differentiation and promotes cell senescence in AS-osteoprogenitor cells.

### WNT16 treatment facilitated cell senescence of AS-osteoprogenitor cells under H_2_O_2_ stimulation

**Fig. 3:**
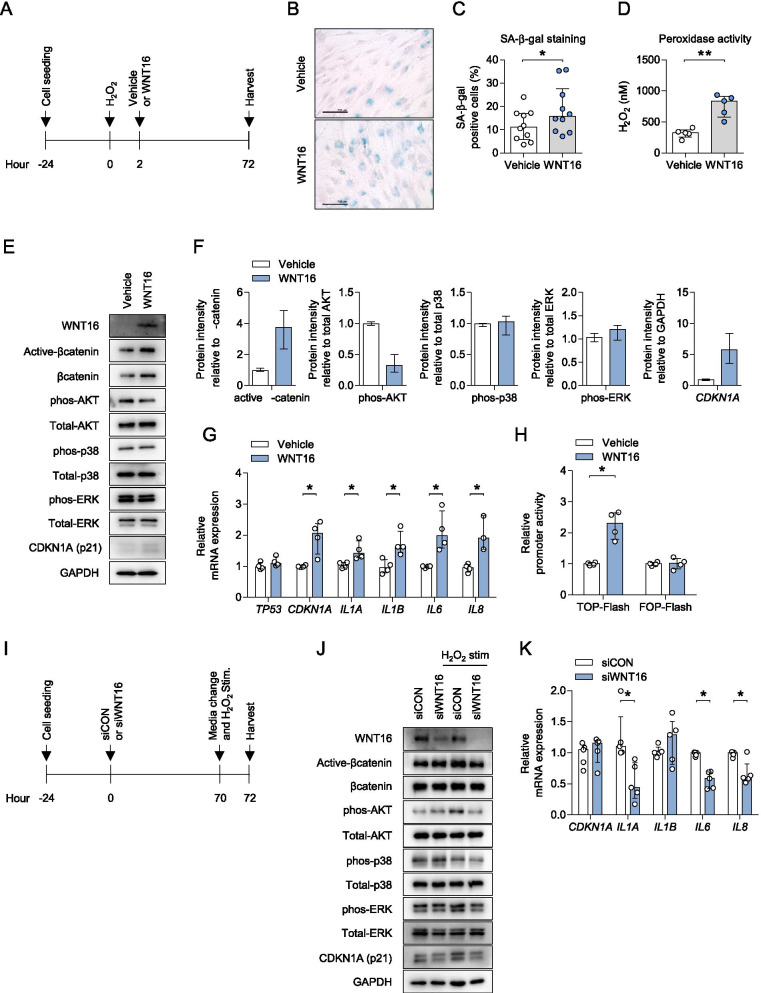
WNT16 treatment facilitated cell senescence of AS-osteoprogenitor cells under H_2_O_2_-stimulation. **A** Experimental design for cell senescence treated with WNT16. AS-osteoprogenitor cells were exposed to H_2_O_2_ for 2 h and then incubated with vehicle or WNT16 treatment for 70 h (*n*=5 per each group). Analysis of **B** SA-β-gal staining, **C** counting results of SA-β-gal-positive cells, **D** assessment of osteoprogenitor cell lysates using hydrogen peroxidase assay (*n*=5 per each group), **E** immunoblotting for protein level, **F** protein quantification of Fig. 3**E**, and **G** RT-qPCR for mRNA level (*n*=4 per each group). **H** SaOS2 cells were transfected with TOP or FOP-flash in the presence β-galactosidase for 24 h, incubated with WNT16 treatment for 24 h, and then analyzed by luciferase assays (*n*=4 per each group). **I** Experimental design for cell senescence treated with siRNA against WNT16. AS-osteoprogenitor cells were transfected with 100 nM WNT16 siRNA #2 for 70 h, exposed to H_2_O_2_ for 2 h, and followed by analysis of **H** immunoblotting for WNT16 protein level and **I** qPCR for mRNA level (*n*=5 per each group). Data are presented as the median and interquartile range. *p<0.05, **p<0.01. Representative images are shown. Scale bar = 200 μm

We next sought to investigate AS without osteogenic differentiation and designed an experimental condition using H_2_O_2_-induced senescence (Fig. 3A). WNT16 treatment in AS-osteoprogenitor cells exhibited a marked increase in SA-β-gal staining, SA-β-gal positive cells rates, and hydrogen peroxidase activity (Fig. 3B–D). In these conditions, WNT16 treatment increased active β-catenin, β-catenin, and p21 protein expression in AS-osteoprogenitor cells and decreased phos-AKT without changes in phos-p38 or phos-ERK protein levels (Fig. 3E, F). Moreover, WNT16 treatment significantly increased p21 mRNA expression and senescence-associated secretory phenotype (SASP)-related genes such as IL-1α, IL-1β, IL-6, and IL-8 (Fig. 3G) in AS-osteoprogenitor cells. WNT16 treatment activated the β-catenin functional motif, as confirmed by promoter activity assays (Fig. 3H). In contrast, WNT16 knockdown using siRNA attenuated the increased phos-AKT and CDKN1A proteins by H_2_O_2_ stimulation (Fig. 3J). WNT16 knockdown groups significantly reduced the production of SASP mRNA expression response to H_2_O_2_ stimulation (Fig. 3K). Taken together, WNT16 treatment facilitated cell senescence of AS-osteoprogenitor cells under H_2_O_2_ stimulation.

## Discussion

In this study, WNT16 was significantly increased in AS-osteoprogenitor cells and was observed in AS facet joint tissues. During osteoblast differentiation, WNT16 treatment in AS-osteoprogenitor decreased matrix maturation and mineralization accompanied by increased cell senescence, but not in control-osteoprogenitor. Moreover, WNT16 treatment strikingly facilitated cell senescence, p21 protein, and SASP expression in AS-osteoprogenitor cells under H_2_O_2_-induced stimulation, while WNT16 knockdown attenuated this senescence effect. Collectively, high WNT16 expression in AS-osteoprogenitor cells revealed that inhibiting bone mineralization and inducing cellular senescence of AS-osteoprogenitor, indicating that it might influence the bone loss in patients with AS.

In general, WNT3 is well known to play a positive role in bone formation through β-catenin activation. Li et al. showed that WNT molecule serum levels (WNT3a, WNT4, WNT5a, WNT7b, and WNT10) were increased dependent on the progression status of spinal ankylosis. In particular, the result showed that WNT3 and WNT5 were associated with hyper-osteoblastic activity and spinal ectopic new bone formation, and their expressions were in response to TNF stimulation [3]. In this paper, we showed that WNT3 expression was highly expressed in AS-osteoprogenitor cells (Fig. 1A, B). However, reports of WNT3 serum level in AS groups have been inconsistent [3, 29, 30], suggesting that the functional and pathophysiological changes need further investigation and a constructive approach.

Many studies have indicated that WNT16 transgenic mice show an increase in bone mineral density and mass whereas WNT16 knockout mice show a decrease in cortical and cancellous bone mass [14–16]. Here, we suggest that human WNT16 plays a paradoxical role in bone formation in AS. Consistent with our finding, DKK1 knockdown in osteoblast of WNT16 transgenic mice showed the reduction of calcified nodule formation relative to the control [31], indicating that the functional role of WNT16 in mineralization may be different depending upon DKK1 expression. Also, human meta-analyses have reported low levels of DKK1 in AS [32], and high WNT16 expression in AS is considered to lead to restrained bone mineralization.

Many studies have reported that WNT16 expression is strongly associated with bone mineral density, suggesting that WNT16 is a therapeutic target for bone loss-related diseases such as osteopenia or osteoporosis. While these data are mainly from murine models, there are a few human study results. It is a questionable issue whether WNT16 plays a functional role in humans. Since cell senescence in old mice exhibits a decline in bone mass accompanied by Osterix protein decrease [33], we analyzed whether WNT16 treatment affects Osterix expression. However, we found no change in Osterix mRNA expression (data now shown).

WNT16 genome-wide association study (GWAS) meta-analyses have shown that polymorphisms of the WNT16 locus are strongly associated with cortical bone thickness, BMD, and risk of osteoporosis fracture in humans [15, 34–38]. Therefore, these studies indicate that WNT16 is an important determinant of cortical bone mass and risk of fracture. As discussed above, the functional relevance of WNT16 in the regulation of the bone and mineralization has been confirmed in both in vitro and in vivo studies. Importantly, there is a lack of information on the functional role of WNT16 in AS-associated bone loss; therefore, further study on the relationship between bone loss and WNT16 locus polymorphisms in patients with AS is needed.

We measured WNT16 levels in collected serum and confirmed that there was no significant difference between the control and AS groups (data now shown). Our serum results are consistent with previous reports [3], but WNT16 expression was high in AS-osteoprogenitor cells and AS bone tissue compared to the control. Therefore, the difference in WNT16 level between serum and pathological tissue can be interpreted as the distinction between systemic and local disease.

A limitation of our paper is that we did not provide the direct regulatory mechanisms on WNT16-induced p21 expression in AS-osteoprogenitor cells. Second, we did not explore that how WNT16 increases oxidative stress and related protein expression in AS-osteoprogenitor cells. Third, further experiments are needed to explore the effect of WNT16 treatment on the reduction of bone formation-related genes.

## Conclusion

Our present study shows that WNT16 is highly expressed in AS-osteoprogenitor cells and in destructive bone tissue. WNT16 treatment in AS-osteoprogenitor cells inhibits mineralization of osteoblast differentiation accompanied by promoting cell senescence. Therefore, elevated WNT16 might be a putative indicator for bone loss in AS.

## Supplementary Information

**Additional file 1: Supplementary Figure 1**. The overall image of a well for 96 well plates and its enlarged changes by Nikon microscopy.

**Additional file 2: Supplementary Figure 2**. The result of siRNA efficiency test. (A) Total four human osteoblasts cell lines (MG63, FOB, SaOS2, U2OS) were extracted total RNA and analyzed by RT-PCR to test WNT16 level. (B) FOB cell were transfected with five siRNA oligo against human WNT16 and analyzed by RT-PCR for siRNA efficiency.

**Additional file 3: Supplementary Figure 3**. Analysis of WNT16 level in collected sera. The human WNT16 levels were measures in the collected sera (HC, n=3; AS, n=36) using ELISA. N.S., not significant.

**Additional file 4: Supplementary Figure 4**. WNT16 treatment inhibited ALP activity in AS-osteoprogenitors. AS-osteoprogenitors were plated in 96 wells plate and treated with 0, 25, or 50 ng/ml WNT16, and then analyzed as indicated by (A) WST assay for cell proliferation. (B) ALP and COL staining, and (C) ALP activity. Data are presented as the mean ± SD (n=3). *p<0.05.

## Data Availability

Microarray day used in this study are available from the corresponding author upon reasonable request.

## Abbreviations

AS: Ankylosing spondylitis
HC: Healthy controls
ALP: Alkaline phosphatase
VON: Von Kossa
HA: Hydroxyapatite
OSE: Osteoblast-specific element
BSP: Bone sialoprotein
OPN: Osteopontin
OCN: Osteocalcin
CASP3: Caspase 3
SASP: Senescence-associated secretory phenotype
mSASSS: Modified Stoke Ankylosing Spondylitis Spinal Score
CRP: C-reactive protein
OS: Oxidative stress
ROS: Reactive oxygen species
MSC: Mesenchymal stem cells
