# Supplementary figures and images for: WNT16 elevation induced cell senescence of osteoblasts in ankylosing spondylitis
Source: Arthritis Res Ther. 2021 Dec 8;23:301. doi: 10.1186/s13075-021-02670-0 (PMC8653593; doi:10.1186/s13075-021-02670-0)

**
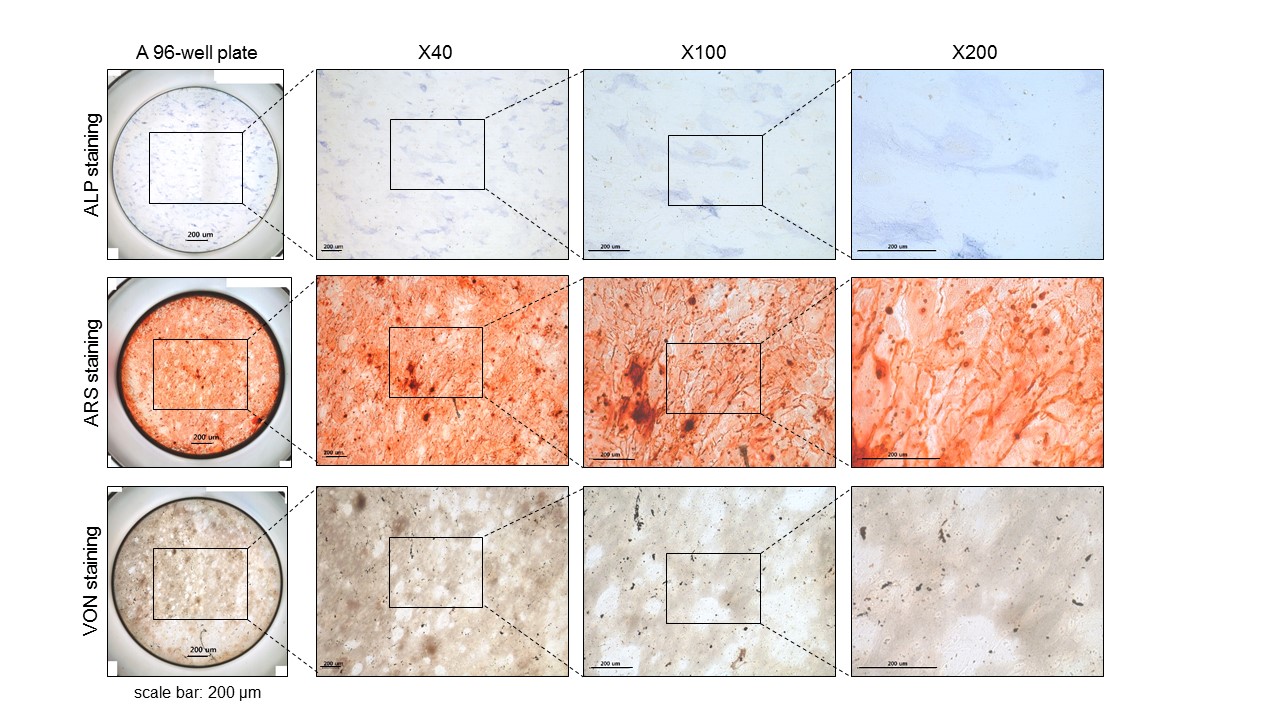
**

Supplement: Supplementary file 1 — Additional file 1: Supplementary Figure 1. The overall image of a well for 96 well plates and its enlarged changes by Nikon microscopy. [file 13075_2021_2670_MOESM1_ESM.docx]

**
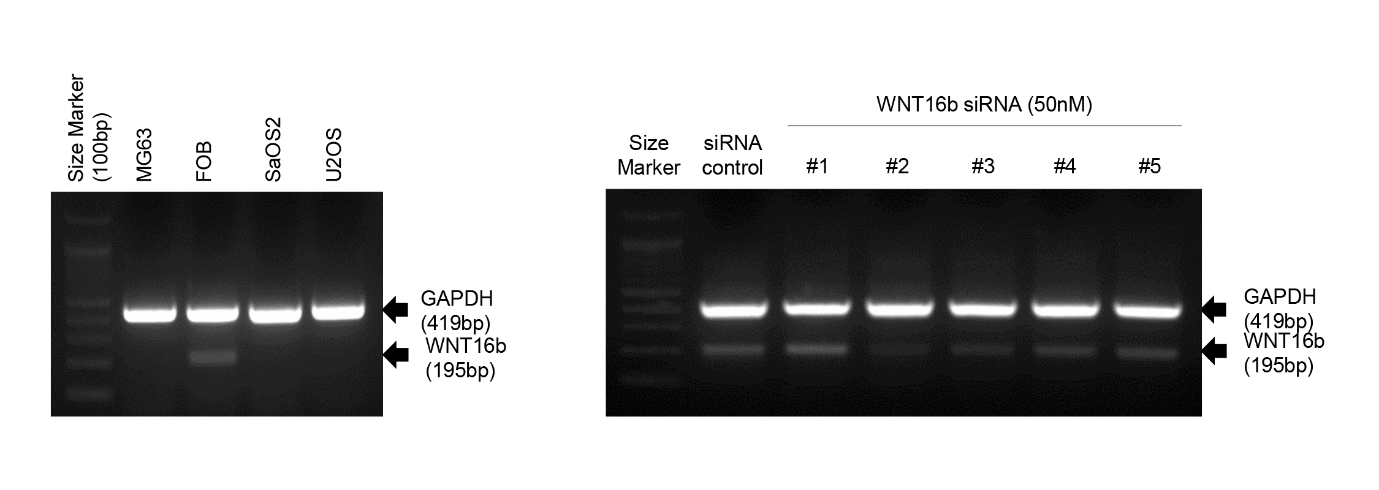
**

Supplement: Supplementary file 2 — Additional file 2: Supplementary Figure 2. The result of siRNA efficiency test. (A) Total four human osteoblasts cell lines (MG63, FOB, SaOS2, U2OS) were extracted total RNA and analyzed by RT-PCR to test WNT16 level. (B) FOB cell were transfected with five siRNA oligo against human WNT16 and analyzed by RT-PCR for siRNA efficiency. [file 13075_2021_2670_MOESM2_ESM.docx]

**
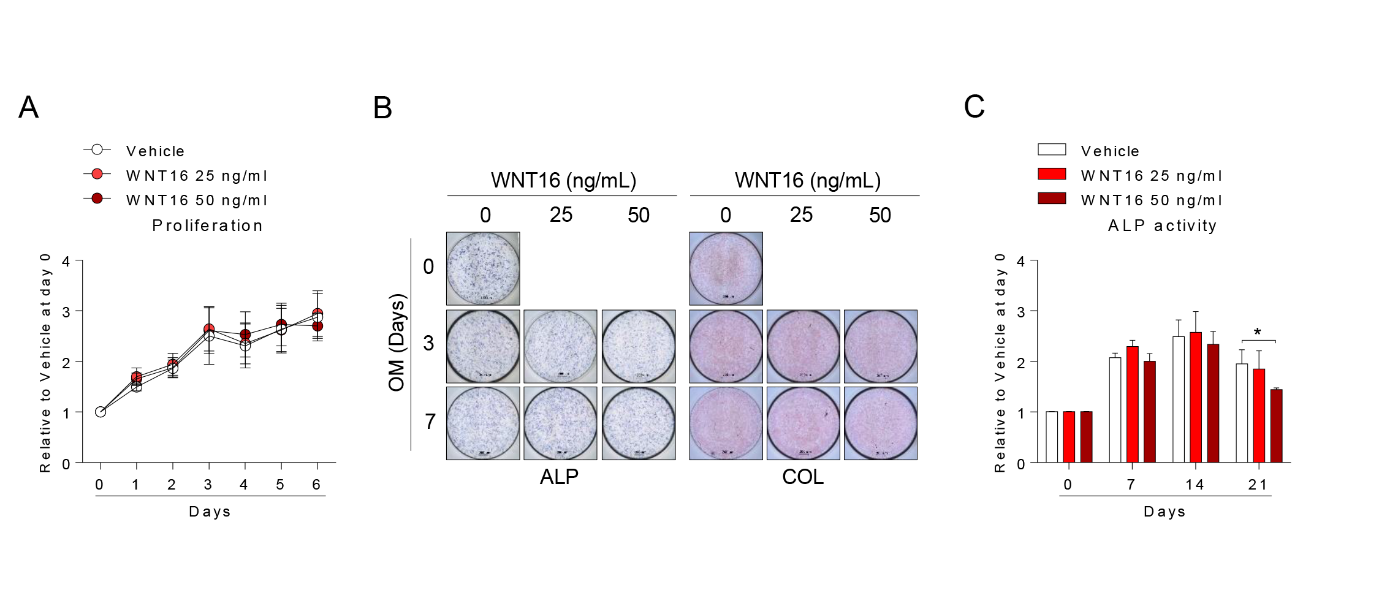
**

Supplement: Supplementary file 4 — Additional file 4: Supplementary Figure 4. WNT16 treatment inhibited ALP activity in AS-osteoprogenitors. AS-osteoprogenitors were plated in 96 wells plate and treated with 0, 25, or 50 ng/ml WNT16, and then analyzed as indicated by (A) WST assay for cell proliferation. (B) ALP and COL staining, and (C) ALP activity. Data are presented as the mean ± SD (n=3). *p<0.05. [file 13075_2021_2670_MOESM4_ESM.docx]
