# Supplementary material for: WNT16 elevation induced cell senescence of osteoblasts in ankylosing spondylitis
Source: Arthritis Res Ther. 2021 Dec 8;23:301. doi: 10.1186/s13075-021-02670-0 (PMC8653593; doi:10.1186/s13075-021-02670-0)
Supplement: Supplementary file 2 [file 13075_2021_2670_MOESM3_ESM.docx]

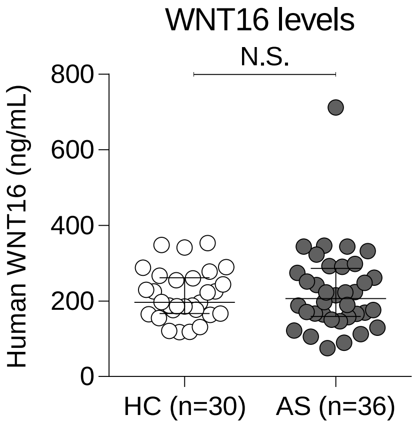
WNT16 levels
N.S.
Human WNT16 (ng/mL)
800
600
400
200
0
HC (n=30)
AS (n=36)
